# Cyclosporine H Improves the Multi-Vector Lentiviral Transduction of Murine Haematopoietic Progenitors and Stem Cells

**DOI:** 10.1038/s41598-020-58724-x

**Published:** 2020-02-04

**Authors:** Leonid Olender, Nir Bujanover, Omri Sharabi, Oron Goldstein, Roi Gazit

**Affiliations:** The Shraga Segal Department of Microbiology, Immunology, and Genetics, Faculty of Health Sciences, National Institute for Biotechnology in the Negev, Ben-Gurion University of the Negev, Beer Sheva, 84105, Israel

**Keywords:** Adult stem cells, Haematopoietic stem cells, Stem-cell research

## Abstract

Haematopoietic stem cells (HSCs) have the potential for lifetime production of blood and immune cells. The introduction of transgenes into HSCs is important for basic research, as well as for multiple clinical applications, because HSC transplantation is an already established procedure. Recently, a major advancement has been reported in the use of cyclosporine H (CsH), which can significantly enhance the lentivirus (LV) transduction of human haematopoietic stem and progenitor cells (HSPCs). In this study, we employed CsH for LV transduction of murine HSCs and defined haematopoietic progenitors, confirming previous findings in more specific subsets of primitive haematopoietic cells. Our data confirm increased efficiencies, in agreement with the published data. We further experimented with the transduction with the simultaneous use of several vectors. The use of CsH yielded an even more robust increase in rates of multi-vector infection than the increase for a single-vector. CsH was reported to reduce the innate resistance mechanism against LV infection. We indeed found that additional pretreatment could increase the efficiency of transduction, in agreement with the originally reported results. Our data also suggest that CsH does not reduce the efficiency of transplantation into immune-competent hosts or the differentiation of HSCs while enhancing stable long-term expression *in vivo*. This new additive will surely help many studies in animal models and might be very useful for the development of novel HSC gene therapy approaches.

## Introduction

Haematopoietic stem cells (HSCs) are the source for all blood and immune cells in an adult organism^1^. HSC transplantation is among the main clinical applications of stem cells^2^. Therefore, manipulation of HSCs for experimental research is of high interest, and clinical utilization of these cells for gene therapies is of acute need^3^. The introduction of the transgene may complement either a missing or a mutated gene, allowing functional manipulations of the blood and immune systems in a practical and efficient way due to the potency of HSCs. Lentiviruses (LVs) have been demonstrated to integrate into the HSC genome and sustain transgene expression over a prolonged time following transplantation^4–6^. Nevertheless, rates of transduction and the ability to sustain HSC potency following *ex vivo* manipulation still pose major obstacles. The number of primary HSCs is a limiting factor, as these are rare cells estimated to account for a few thousand of the cells in a single mouse^7^ or 50,000–200,000 of the cells in an adult human^8^. Therefore, any improvement in the efficiency of LV transduction in primary HSCs is of interest, as long as it does not lead to impairment of their long-term multilineage repopulation capacity upon transplantation.

The interest in the introduction of transgenes into HSCs is reflected by the plethora of studies reporting various vectors and strategies. Importantly, as HSCs are defined by their long-term potency, in this study, we focused on vectors providing long-term expression, while other vectors might be of use for transient expression^9^. Classically, using retrovirus- or lentivirus-based vectors has been reported to obtain stable expression in HSCs and their progeny following transplantation^3^. However, such an experimental setting has also encountered difficulties in gaining high frequencies of transgene-expressing cells^3^, and it is known that using high levels of viruses can have a deleterious impact on the viability and potency of these cells upon transplantation^5^. Other vectors used for transgene delivery into HSCs include transposons^10^, episomes^11^, and adeno-associated virus 6^12^. Although some publications have suggested direct delivery of DNA into HSCs using electroporation^13^, this approach did not yield highly effective protocols. The recent utilization of CRISPR appears to be very promising in the context of HSCs, as any manipulation of these cells can be directly used for clinical applications, and there are a number of candidate genes to manipulate^14,15^. The ability to efficiently deliver transgenes into HSCs without affecting their long-term multilineage repopulation capacity could benefit many current and future studies in the field.

Both basic research and possible clinical applications involving genetically modified cells rely heavily on the ability to develop reproducible protocols with adequate readouts and outcomes. It is occasionally possible to gain a proof-of-concept with only a handful (a few percent or even less) of transgene-positive cells in which the readout is significantly distinct from the background levels. However, having a low transduction efficiency is not only frustrating but also can be prohibitive if the starting population of cells is limited. Bona-fide functional HSCs make up a very rare population in the bone marrow (BM), estimated at 1 in every 50,000 cells or even less in an adult mouse^16,17^. Importantly, we have solid evidence that only these HSCs bear true life-long potency, while other primitive haematopoietic cells are active only for a limited amount of time^18–20^. Multiple attempts have been made to overcome the limitations of HSC numbers by either *ex vivo* expansion^21,22^ or various reprogramming strategies using pluripotent^23^, endothelial^24,25^ or blood cells^26^. All of these are essentially limited by the low efficiency of *ex vivo* manipulations of HSCs or Progenitors. On the other hand, primary HSCs are readily available as either allogeneic or even autologous cells that have been clinically established for efficient HSC transplantation, saving tens of thousands of lives every year^27^. Thus, increasing the efficiency of LV transduction in HSCs is clearly of an acute need.

LV vectors have been developed and improved over the last 30 years^28^. They are able to transduce the vast majority of cell types, with VSVG (vesicular stomatitis virus G-protein) pseudo-typing providing avidity to virtually all types of cells^29^. The ability to integrate into the genome of non-dividing cells has turned LVs into a versatile and abundant tool for research and development in various gene therapy approaches. Nevertheless, mammalian cells have evolved to resist viral infection, and there are multiple mechanisms by which cells can block viral entry, activity, and integration^30^. The immune system acts to protect our body against all pathogens, including viruses, and there are immune cells that may have increased specialized antiviral functions^31^. Among the mechanisms reported to resist LV transduction, type-I interferons (IFNα and IFNβ) provide a major pathway integrating danger signals and limiting viral spread among cells^32^. HSCs are known to respond to type-I IFN^33–37^. Clearly, any ability to overcome cellular resistance mechanisms may provide improved transduction and may further protect cells from subsequent damage and loss of potency. Immunosuppressive drugs target some of the innate mechanisms that also play an essential role in the antiviral response^38^.

Cyclosporins are a group of compounds originally isolated from fungi^39,40^ and are known primarily as immunosuppressive drugs used in transplantation and autoimmune diseases^41^. Cyclosporine A was the first compound of this group reported to enhance LV transduction into HSCs^42^, in contrast with its antiviral effect on differentiated cells^43^. This might be related to the unusual induction of proliferation in HSCs following IFNα stimulation in contrast with the induction of cell cycle arrest in other cell types^33,34,37^. Recently, cyclosporine H (CsH) was reported by Petrillo and Kajaste-Rudnitski to significantly enhance LV transduction into human HSPCs^44^. CsH is a metabolite of cyclosporine A. CsH is not immunosuppressive and has been reported to act through interferon-induced transmembrane protein 3 (IFITM3)^44^. Intriguingly, this finding is in agreement with studies showing that HSPCs express an interferon signature genes even in the naive state^33,34,37^. Clearly, interferon response genes can be further upregulated upon stimulation that may occur during the isolation and *ex vivo* manipulation of the cells. Petrillo *et al*. demonstrated how CsH improved methods using LV transduction into human HSPCs^44^, including functional xenotransplantations into immune-deficient mice.

Hereby, we used CsH to transduce murine HSCs or defined progenitors with single and multiple LV vectors. First, we observed enhanced transduction *in vitro*, in agreement with the findings of Petrillo and Kajaste-Rudnitski^44^. This was true for highly purified HSCs, as well as for granulocyte-monocyte progenitors (GMPs). Transducing cells with three types of LVs gained even more robust enhancement of multi-vector infection than the enhancement of a single vector. Pretreatment of primitive haematopoietic cells further increased the transduction rates of single and multiple vectors but resulted in decreased expansion *in vitro*. Finally, we demonstrated that LV-transduced murine HSCs treated with CsH retain their ability to be transplanted into syngeneic immune-competent mice while sustaining multi-vector expression.

## Materials and Methods

### Isolation of primary stem cells and progenitors

Primary cells were extracted from the femora and tibiae of C57BL/6J or B6-rtTA mice (JAX 6965) and enriched using the Histopaque-1083 separation reagent (Sigma-Aldrich, St. Louis, MO, USA). Cells were then stained with the following antibodies: lineage cocktail (CD3 – clone 17A2, Ly-6G/Ly-6C – clone RB6-8C5, CD11b – clone M1/70, CD45R/B220 – clone RA3-6B2, and TER-119 – clone Ter-119), cKit (CD117)-APC-Cy7 (clone 2B8), Sca1 (Ly-6A/E)– APC (clone D7), CD150 (SLAM)– PE-Cy7 (clone TC15-12F12.2), CD48-PerCP-Cy5.5 (clone HM48-1), CD34-FITC (clone RAM34) and CD16/32-PE (clone 93). Populations of interest were sorted using a FACS-Aria III (BD Biosciences, San Jose, CA, USA).

### Primary cell culture

Primary cells were cultured in Biotarget-1 serum-free medium supplemented with 2% L-glutamine, 1% penicillin-streptomycin, 1 mM sodium pyruvate, 1% nonessential amino acid solution (all from Biological Industries, Beit Haemek, Israel), 0.06 mM 2-mercaptoethanol (Sigma-Aldrich) and 2 ng/ml doxycycline. The cytokines (PeproTech, Rehovot, Israel) that were added to the growth medium were as follows: murine SCF, murine TPO, murine IL-3 and murine FLT3L (all 10 ng/ml).

### LV transduction in the presence of CsH

Sorted primary cells were cultured overnight in medium as described above. Transduction with VSVG pseudotype LVs was performed 24 hours later. CsH (final concentration 8 µM, unless stated otherwise) or DMSO was added immediately after cell sorting or simultaneously with the LVs and removed after transduction. For spinfection (spinoculation), cells were centrifuged at 1,200 g for 25 minutes at RT. Following centrifugation, the medium was replaced with fresh medium containing CsH or DMSO, and the cells were incubated for an additional 24 hours in the presence of these compounds prior to transplantation into lethally irradiated recipients.

### Flow cytometric analysis

Cells were washed and resuspended in PBS supplemented with 2 mM EDTA and 2% FCS (foetal calf serum). PI (propidium iodide) or DAPI was used for the detection of viable cells. The fluorescence intensities of individual cells were measured using a Gallios flow cytometer (Beckman-Coulter, Brea, CA, USA) or a NovoCyte flow cytometer (ACEA Biosciences, San Diego, CA, USA). Data were analysed and visualized using Kaluza analysis software (Beckman-Coulter).

### Mice, transplantation, and bleeding

All experiments involving mice were carried out according to the ethical guidelines following the approval of the Ben-Gurion University and of the Israel Animal Care and Use Committees (approvals #IL-27-05-2013 and #IL-01-01-2017). Congenic F1 (CD45.1/2) or CD45.1 (JAX 2014) recipients were lethally irradiated prior to transplantation. LV-transduced cells were injected intravenously together with whole BM CD45.1 or F1 competitor cells. Peripheral blood was taken from the tail at designated time points, and lysis of red blood cells was performed using ACK lysis buffer. Cells were stained with CD45.2-Pacific Blue and CD45.1-PE/Cy7 antibodies and analysed using a Gallios flow cytometer (Beckman-Coulter).

## Results

### CsH enhances the LV transduction of murine BM HSCs and progenitors

**Figure 1:**
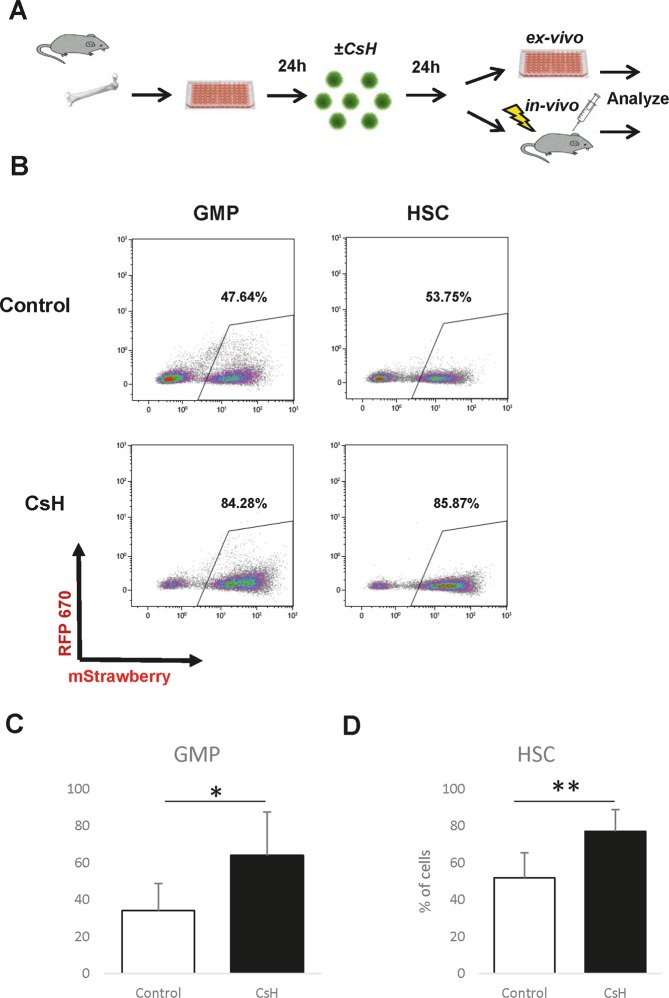
CsH increases the efficiency of lentiviral transduction of murine HSPCs *in vitro*. HSCs (hematopoietic stem cells; defined here as LSK^+^CD48^−^CD150^+^CD34^low^ cells) or GMPs (granulocyte-monocyte progenitors; defined here as LK^+^CD34^+^CD16/32^hi^ cells) were isolated from murine BM, transduced with LVs designed to express a fluorescent reporter (HSCs – MOI 190; GMPs – MOI 100), incubated in the presence of LVs and CsH/DMSO for 24 hours, and cultured for 7 days. The percentage of cells expressing the fluorescent reporter was determined using flow cytometry. (**A**) Schematic representation of the experimental settings.(**B**) Representative FACS plots of transduced GMPs (left panels) or HSCs (right panels) incubated in the presence of DMSO (top panels) or CsH (bottom panels). The percentage of positive cells was quantified relative to uninfected controls (not shown). Data were collected from three independent experiments (n = 3), and triplicate technical replicates were used within the experiments. (**C**,**D**) Quantification of transduction efficiency for GMPs (**C**) and HSCs (**D**); the results are shown as the means ± SDs. Statistical significance was calculated by an unpaired t-test. *p ≤ 0.05, **p ≤ 0.01.

In order to quantify the effect of CsH on the LV transduction of primitive murine haematopoietic cells, we purified granulocyte-monocyte progenitors (GMPs, Lin^−^Sca1^−^cKit^+^CD34^+^CD16/32^hi^ population) and HSCs (Lin^−^Sca1^+^cKit^+^CD48^−^CD150^+^CD34^−/low^ cells) from the bone marrows of C57BL/6 mice and transduced them with an LV expressing fluorescent reporter (Fig. 1A). CsH or DMSO as a control was added at the time of transduction. The expression of the fluorescent reporter was assessed using flow cytometry after a week of culture. In agreement with previously published data, CsH significantly improved the efficiency of vector delivery (Fig. 1B–D)^44^.

### Multi-vector infection further benefits from the use of CsH

**Figure 2:**
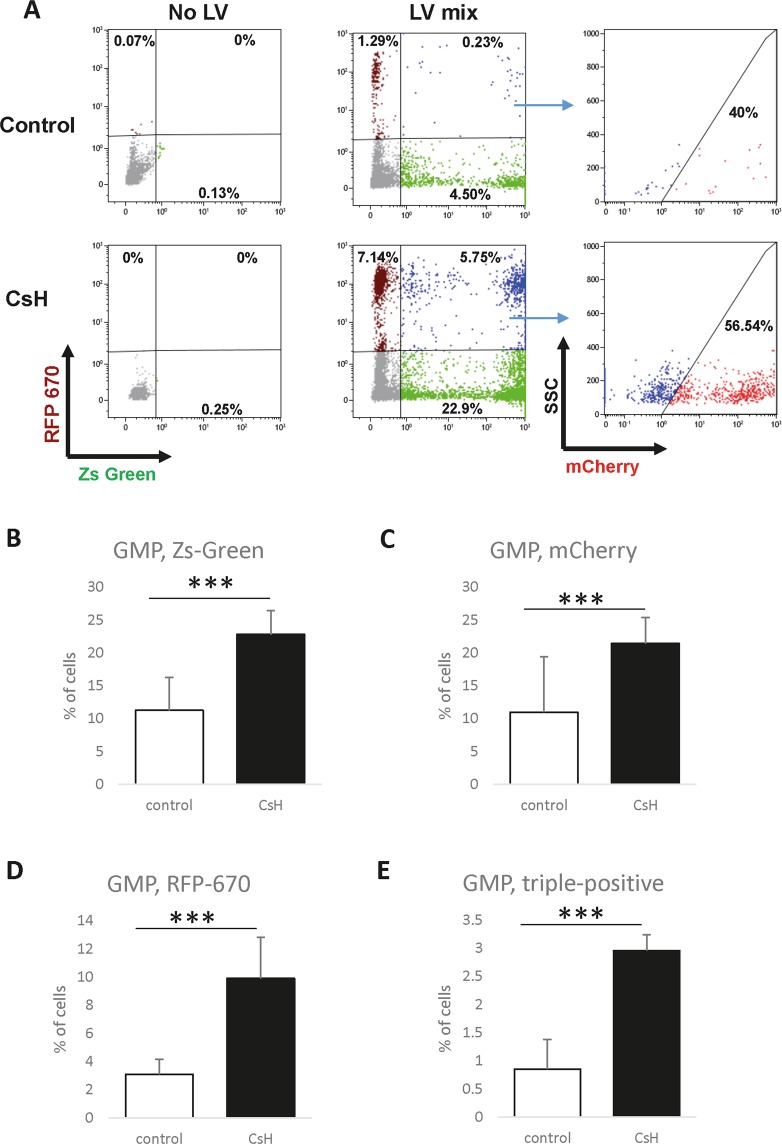
CsH increases the efficiency of multi-vector transduction of primitive murine GMPs *in vitro*. GMPs were sorted from murine BM, transduced with a mix of LVs designed to express ZsGreen, mCherry or RFP670 fluorescent reporters, incubated with LVs and CsH/DMSO for 24 hours, and cultured for 4 days. The percentage of cells expressing fluorescent reporters was determined using flow cytometry. (**A**) Representative FACS plots for reporter-derived fluorescence in uninfected (no LVs, left) and triple-infected (LV mix) cells. ZsGreen and RFP670 were gated first (middle plots), and then the expression of mCherry in the ZsGreen^+^RFP^+^ population was assessed (right plots). The results from DMSO-treated controls and samples treated with CsH are shown in the top and bottom plots, respectively. Separate plots showing the total fraction of mCherry-positive cells are presented in Supplementary Fig. 3. Data were collected from four independent experiments (n = 4), and triplicate technical replicates were used within the experiments. (**B–E**) Quantification of each single colour (**B–D**) and of the triple-transduced cells (**E**). MOI values: ZsGreen – 30, mCherry – 25, and RFP670–15. The results are presented as the means ± SDs. Statistical significance was calculated using an unpaired t-test. ***p ≤ 0.001.

**Figure 3:**
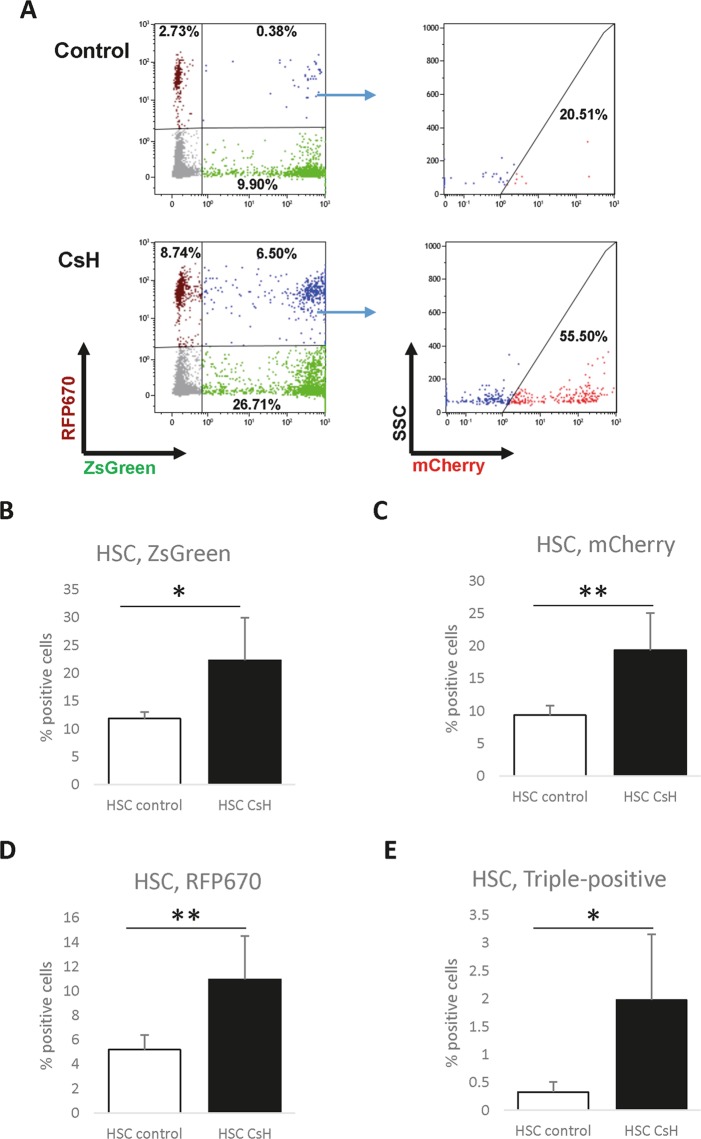
CsH increases the efficiency of multi-vector transduction of murine HSCs *in vitro*. HSCs were sorted from murine BM, transduced with a mix of LVs designed to express ZsGreen, mCherry or RFP670 fluorescent reporters, incubated with LVs and CsH/DMSO for 24 hours, and cultured for 4 days. The percentage of cells expressing fluorescent reporters was determined using flow cytometry (**A**) Representative FACS plots for reporter-derived fluorescence in uninfected (no LVs, left) and triple-infected (LV mix) cells. ZsGreen and RFP670 were gated first (left plots), and then the expression of mCherry in the ZsGreen^+^RFP^+^ population was assessed (right plots). The results from DMSO-treated controls and samples treated with CsH are shown in the top and bottom plots, respectively. Separate plots showing the total fraction of mCherry-positive cells are presented in Supplementary Fig. 3. Data were collected from four independent experiments (n = 4), and triplicate technical replicates were used within the experiments. (**B–E**) Quantification of each single colour (**B–D**) and of the triple-transduced cells (**E**). MOI values: ZsGreen – 50, mCherry – 40, and RFP670–25. The results are presented as the means ± SDs. Statistical significance was calculated using an unpaired t-test. *p ≤ 0.05, **p ≤ 0.01.

**Figure 4:**
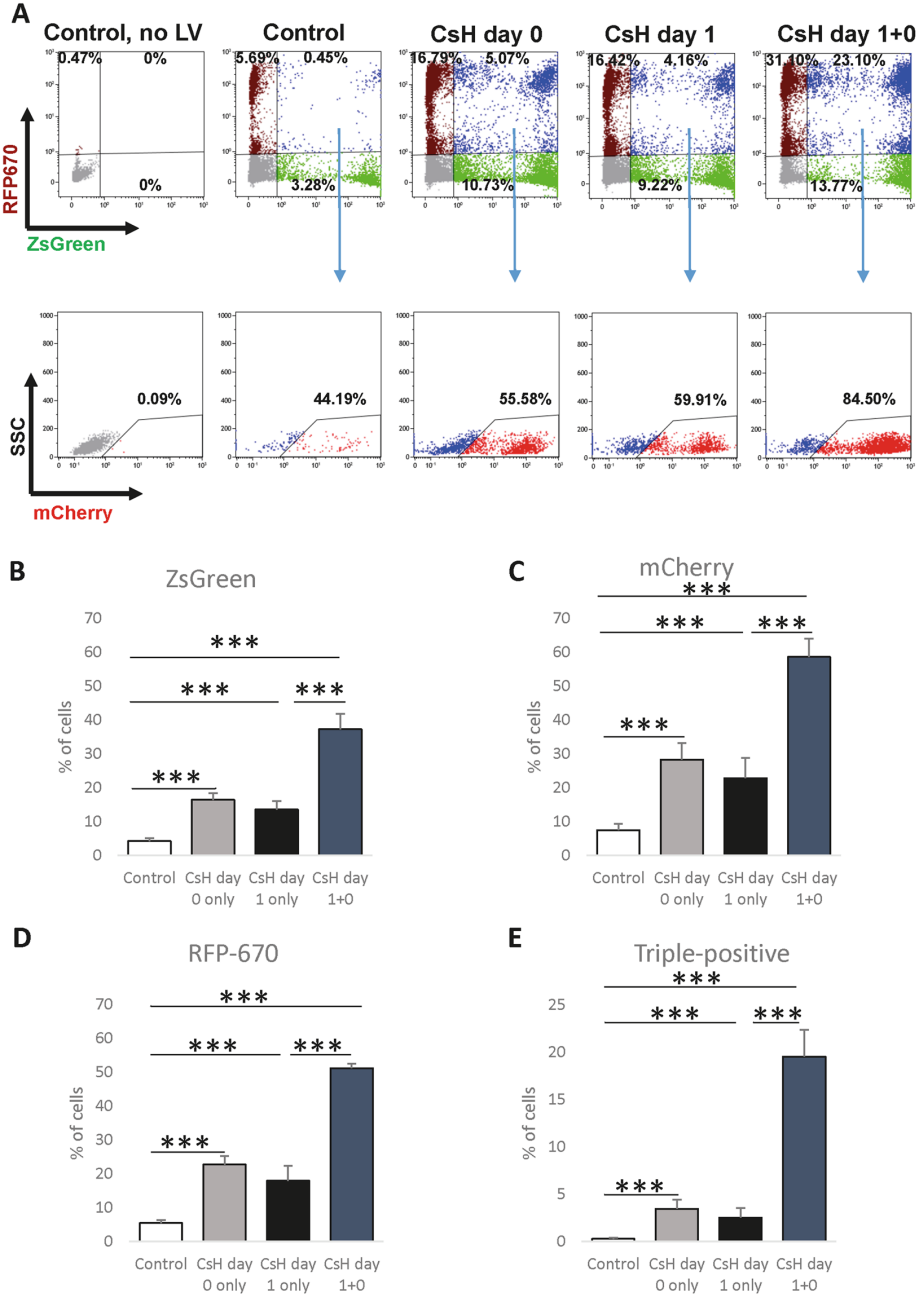
Additional preincubation with CsH further increases the efficiency of multi-vector transduction into primary murine GMPs. GMPs were sorted from murine BM, transduced with a mix of LVs designed to express ZsGreen, mCherry or RFP670 fluorescent reporters, and incubated in the presence of DMSO or CsH for 24 hours. Following the initial incubation, cells were exposed to the mixture of LVs encoding ZsGreen, mCherry or RFP670 fluorescent reporters and incubated with LVs and DMSO/CsH for an additional 24 hours. Cells were cultured for 6 days, and the percentage of cells expressing fluorescent reporters was determined using flow cytometry. (**A**) Representative FACS plots for reporter-derived fluorescence. ZsGreen and RFP670 were gated first (top plots), and then the expression of mCherry in the ZsGreen^+^RFP^+^ population was assessed (bottom plots). Data were collected from four independent experiments (n = 4), and triplicate technical replicates were used within the experiments. (**B–E**) Quantification of each single colour (**B–D**) and of the triple-transduced cells (**E**). MOI values: ZsGreen – 20, mCherry – 25, and RFP670–20. The results are presented as the means ± SDs. Statistical significance was calculated using an unpaired t-test. ***p ≤ 0.001.

We then sought to check the effect of CsH on the simultaneous delivery of several LV vectors into primitive haematopoietic cells. Purified HSCs or GMPs were transduced with a mixture of three LVs simultaneously. Each of the LVs was designed to express ZsGreen, mCherry, or RFP670 fluorescent protein. CsH or DMSO was added during transduction as described above, and the cells were analysed using flow cytometry 4 days later (Figs. 2, 3 and Supplementary Fig. S3). CsH led to a highly significant increase in the expression of each of these reporters (Figs. 2A–D and 3A–D) and, more importantly, increased the percentage of cells expressing all three fluorescent proteins simultaneously from less than 1% to an average of 2–3% in both cell populations (from an average of 0.85% to an average of 2.95% in GMPs and from an average of 0.33% to an average of 1.98% in HSCs, Figs. 2E and 3E). Interestingly, prolonged exposure of GMPs to the chemical resulted in an even more robust increase in the efficiency of LV transduction (Fig. 4) and had a dramatic effect on the rates of multi-vector infection (an increase in the percentages of triple-positive cells to an average of 19.5%, Fig. 4E). We also found that incubation with CsH prior to transduction without subsequent addition of the chemical had similar effects to exposure to CsH simultaneously with the addition of LVs (Fig. 4). However, our results indicate that while benefiting the infection rates, both a higher concentration and prolonged exposure to CsH can significantly decrease the expansion of murine HSPCs (LSK) in culture (Supplementary Fig. S2). Our observations also suggest that higher CsH concentrations might cause acute death of these cells (Supplementary Fig. S1). Taken together, our data show that CsH is a potent facilitator of multi-vector delivery into primary murine HSCs and GMPs, but the protocol should be optimized to achieve sufficient transduction rates while maintaining satisfactory cell numbers and properties.

### CsH-enhanced transduction does not limit transplantation into immune-competent recipients

**Figure 5:**
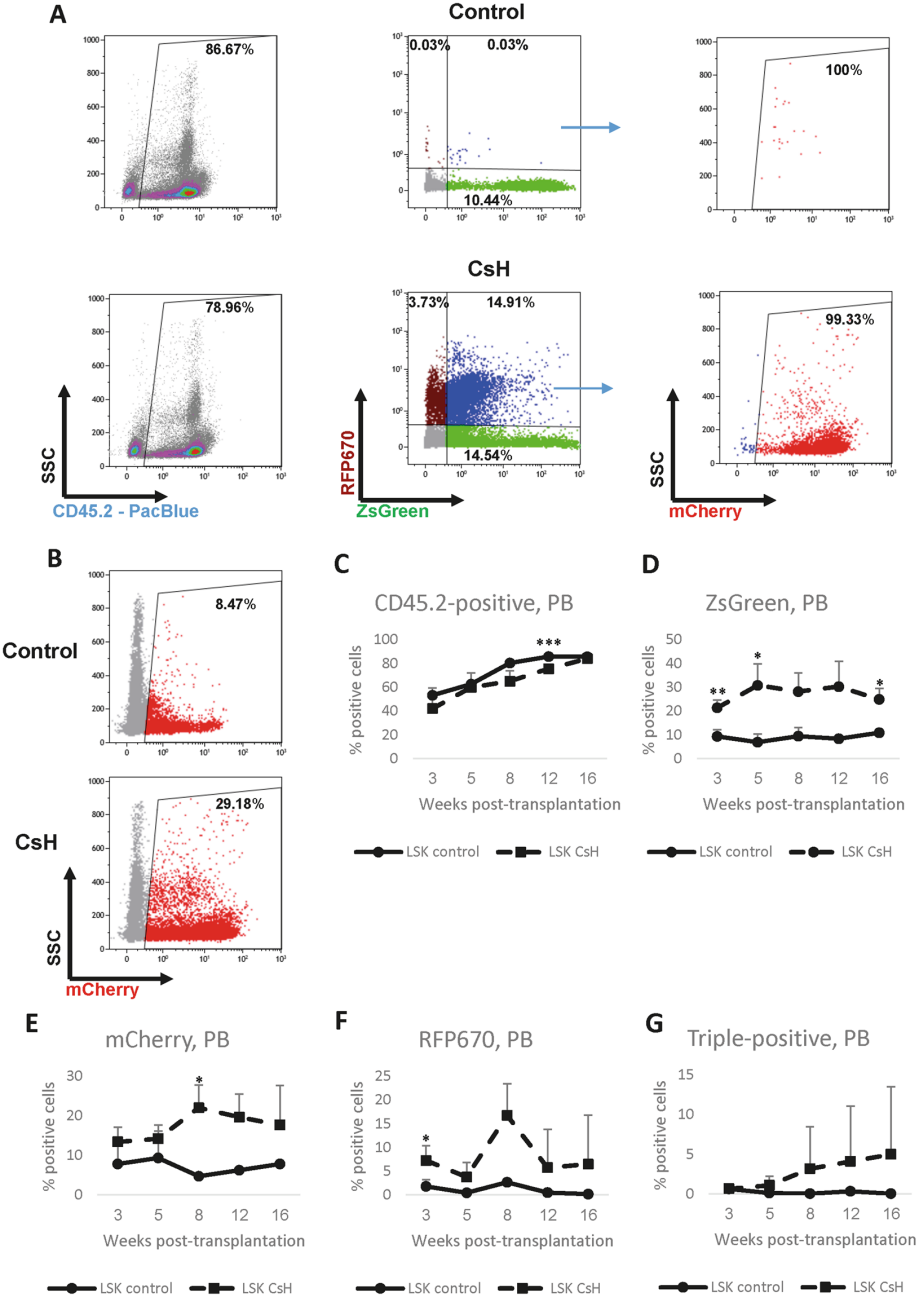
The CsH-mediated increase in the efficiency of LV transduction of primitive murine HSPCs persists through transplantation. LSK (Lin^−^cKit^+^Sca1^+^) cells were isolated from the BM of CD45.2 mice, transduced with a mix of LVs designed to express ZsGreen, mCherry or RFP670 fluorescent reporters using spinoculation (see *Materials and methods*) (MOI values: ZsGreen – 42, mCherry – 28, and RFP670–17), incubated in the presence of CsH/DMSO for 24 hours and injected into lethally irradiated CD45.1 recipients together with whole BM CD45.1 competitor cells (3 mice in each group). The expression of fluorescent reporters and chimerism in the peripheral blood (PB) were assessed at various time points using flow cytometry. (**A**) Representative plots showing the proportion of donor-derived cells and the expression of fluorescent reporters in a mouse from the control group (top panel) vs the CsH group (bottom panel) 16 weeks post-injection. Cells expressing all three fluorescent reporters were identified by assessing the proportion of cells positive for mCherry out of the population of cells positive for both ZsGreen and RFP670. (**B**) The proportion of cells expressing mCherry out of total donor-derived (CD45.2^+^) cells. Data were collected from three independent experiments (n = 3), and triplicate technical replicates were used within the experiments. (**C–G**) Quantification of the percentages of CD45.2^+^ cells (**C**), cells expressing each fluorescent protein (**D–F**), and cells expressing all three reporters (**G**) in the PB of control or CsH mice at various time points post-transplantation. The results are presented as the means ± SDs. Statistical significance was calculated by an unpaired t-test. *p ≤ 0.05, **p ≤ 0.01, ***p ≤ 0.001.

Finally, we wanted to examine whether the effect of CsH on LV transduction into primitive murine haematopoietic cells persists upon transplantation and how the use of the compound affects the functional properties of HSCs *in vivo*. Isolated CD45.2 HSPCs were transduced with a mixture of the three LVs, as described above, in the presence of CsH or DMSO and transplanted into congenic CD45.1 recipients together with CD45.1 whole BM competitor cells. The chimerism and expression of reporters in donor-derived cells from peripheral blood (PB) were assessed at different time points post-transplantation. The total chimerism of CsH-treated HSPCs was similar to that of controls, showing no statistical difference at 16 weeks post-transplantation (Fig. 5C). Importantly, unlike the mice under xenotransplantation settings, congenic immune-competent recipients were used in our experiments. Analysis of LV-derived fluorescence in PB CD45.2^+^ cells revealed that LV transductions with simultaneous CsH administration resulted in higher rates of cells positive for each of the reporters than transductions performed without CsH (Fig. 5A,B,D–F). The triple-positive fraction was also higher in mice that received CsH-treated cells than in the groups that received cells treated with DMSO (Fig. 5G). The elevated frequencies of reporter-positive cells were sustained over time, but often with no statistical significance due to the high variance between animals in each group. The transplantation data demonstrate that the CsH-mediated enhanced LV transduction of murine HSPCs is retained in terms of transgene expression *in vivo*, and using a fine-tuned protocol did not decrease the long-term repopulation potency.

## Discussion

The introduction of a transgene into HSCs is important for research and even more so for clinical application purposes. HSC transplantation can effectively regenerate the blood and immune system for life, so any improvement in the ability to modify these cells *ex vivo* may be directly translated into a benefit for the patients in need. However, many trials have encountered difficulties in genetically manipulating HSCs efficiently^45–47^. Understanding the resistance mechanisms and developing methods to overcome them will help many researchers and impact the broader perspectives of stem cell utilization in regenerative medicine. In this study, we confirmed a recent discovery of CsH as a potent enhancer of LV transduction in HSPCs.

The resistance of HSCs to LV transduction has been reported by multiple studies^42,44,48,49^. Not surprisingly, attempts to increase the efficiency have focused on innate immune mechanisms, and findings related to CsA and rapamycin followed this line of protocols^42,49^. Such protocols had a fine balance between increased transduction rates and sustained potency of the HSCs, as the protocols were ultimately tested in long-term multilineage transplantation settings. Importantly, while *ex vivo* assays may suggest many of the parameters, transplantation is required as an ultimate HSC function test. In addition to viability, phenotypic appearance, proliferation and differentiation potency, there are also issues of homing and interactions with the host immune system. Even with the most recent advanced molecular analysis, there is a critical need for an actual functional assay. Petrillo and colleagues^44^ recently reported that CsH, a metabolic derivative of CsA, increased HSC transduction efficiency while sustaining their potency. We confirmed these findings and extended them into strategies using multi-vector transduction of better-defined stem and progenitor populations. Petrillo *et al*. impressively demonstrated the ability of CsH-treated human HSPCs to engraft immune-deficient mice^44^, and we show that this is also true for murine HSPCs in congenic immune-competent recipients, confirming the ability of CsH-treated cells to sustain their functional properties. HSCs are functionally heterogeneous^50^. The high variance between individually transplanted animals in our data might stem from the heterogeneity in long-term *in vivo* activity of individual HSCs.

Since transduction efficiency is a major concern, we further tested some modifications to the CsH protocol. Our findings suggest that the use of higher concentrations or prolonged exposure to CsH can yield higher transduction rates but might have a cytotoxic effect. Therefore, we highlight the importance of fine-tuning CsH protocols according to specific purposes. Importantly, our data show that pretreatment can significantly enhance the transduction rates, in agreement with the published mechanism by which CsH reduces IFITM3^44^. Quiescence is a hallmark of HSCs, and less metabolically active cells have been reported to have higher transplantation potency^51,52^. Nevertheless, extended exposure to CsH, especially when culture conditions may push the cells to divide, can have a cytotoxic effect. Moreover, some applications may actually require cell division, such as genomic manipulations that rely on homologous recombination mechanisms^53–55^. Therefore, one must be cautious when using CsH according to specific purposes. CsH treatment can be easily limited to the time of viral transduction, possibly as short as one hour if expedited spinfection is used^56^. Interestingly, the anti-proliferative effect of CsH suggests a positive potency-retention effect, as enforced cell division has been correlated with reduced potential in HSCs^57–60^.

HSCs have been at the forefront of research on adult stem cells for over a century^1^. Bone marrow transplantation saves the lives of tens of thousands of patients every year worldwide^2^. However, our limited ability to genetically manipulate HSCs is an obstacle for research and clinical applications. Autologous HSC transplantation with stable transgenes will directly provide cures for several currently untreated conditions, and improved transgenic HSCs will boost research, with a rapid ability to study these adult stem cells and their haemato-immune progeny *in vivo*. We confirmed the findings of a recent publication showing the ability of CsH to enhance HSC transduction with LVs and extend these findings by showing multi-vector expression in murine cells transplanted into congenic immune-competent hosts. This protocol will help many researchers, with further fine-tuning allowing for high transgene expression while sustaining the true potency of adult stem cells.

## Supplementary information

Supplementary Information.

## Data Availability

The authors declare that all data supporting the findings of this study are available within the article or from the corresponding authors upon reasonable request.
