# Supplementary material for: Cyclosporine H Improves the Multi-Vector Lentiviral Transduction of Murine Haematopoietic Progenitors and Stem Cells
Source: Sci Rep. 2020 Feb 4;10:1812. doi: 10.1038/s41598-020-58724-x (PMC7000727; doi:10.1038/s41598-020-58724-x)
Supplement: Supplementary file 1 — Supplementary Information. [file 41598_2020_58724_MOESM1_ESM.pdf]

Cyclosporine H Improves the Multi-Vector Lentiviral Transduction of Murine Haematopoietic Progenitors and Stem Cells

Leonid Olender[1], Nir Bujanover[1], Omri Sharabi[1], Oron Goldstein[1], and Roi Gazit[1]

[1]The Shraga Segal Department for Microbiology, Immunology, and Genetics, Faculty of Health Sciences; National Institute for Biotechnology in the Negev; the Ben-Gurion University of the Negev, Israel POB 84105

Three supplementary figures with captions

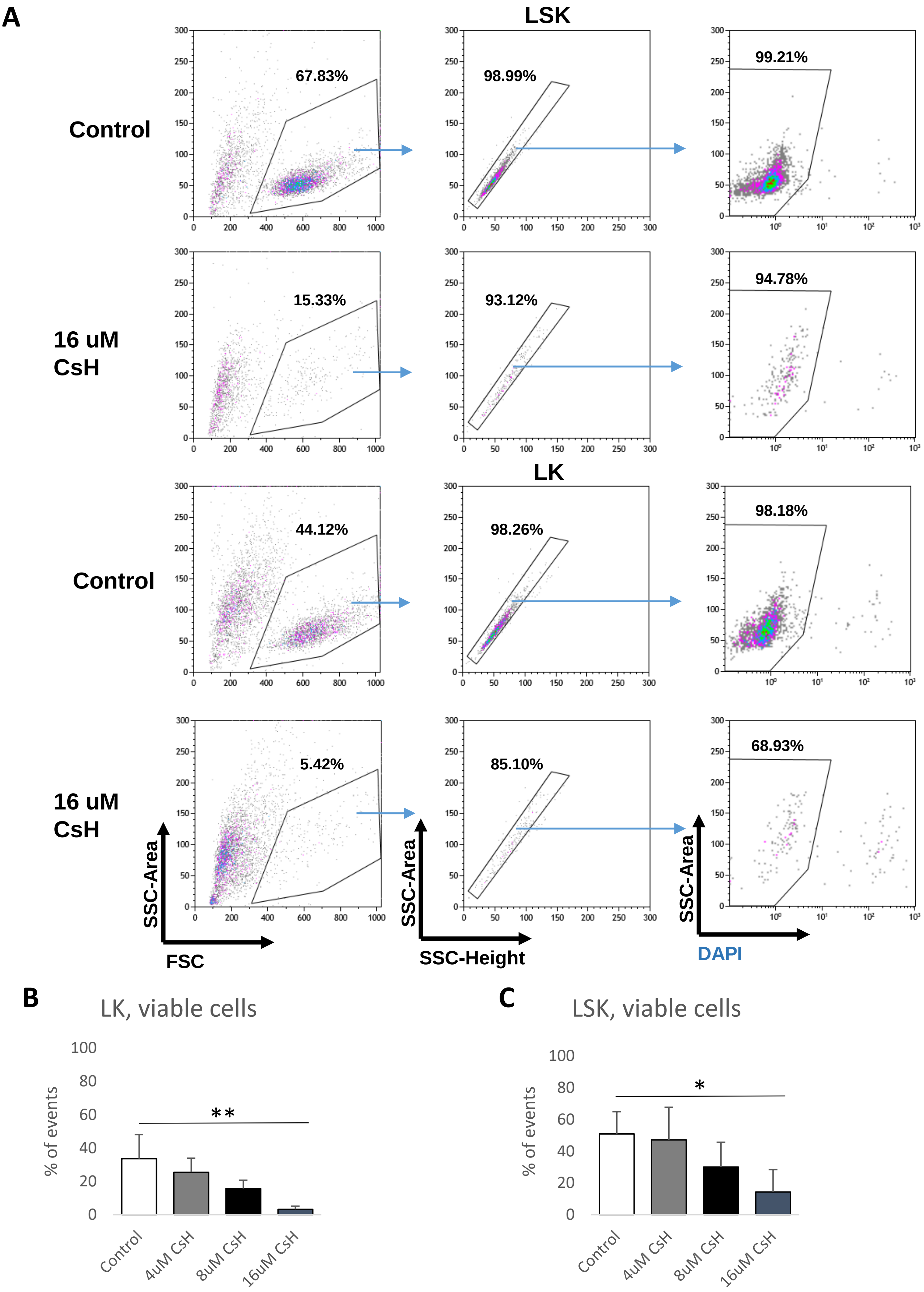

Supplementary Figure S1. Impact of various doses of CsH on the viability of murine HSPCs in culture. LSK (Lin$^-$cKit$^+$Sca1$^+$) and LK (Lin$^-$cKit$^+$Sca1$^-$) cells were sorted from murine BM and incubated for 24 h in medium supplemented with cytokines (see Materials and methods). DMSO (control) or CsH at a gradient of concentrations (4 uM, 8 uM or 16 uM) was added to the medium, and 21 h later, cell viability was assessed with DAPI using flow cytometry. **A.** Representative plots showing the gating strategy and assessment of the viable cell population for control vs 16 uM CsH samples (LSK – upper panels; LK – lower panels). **B** and **C.** Data were collected from four independent experiments (n=4). The means ±SDs are presented. Statistical significance was calculated by an unpaired t-test. *p≤0.05, ** p≤0.01.

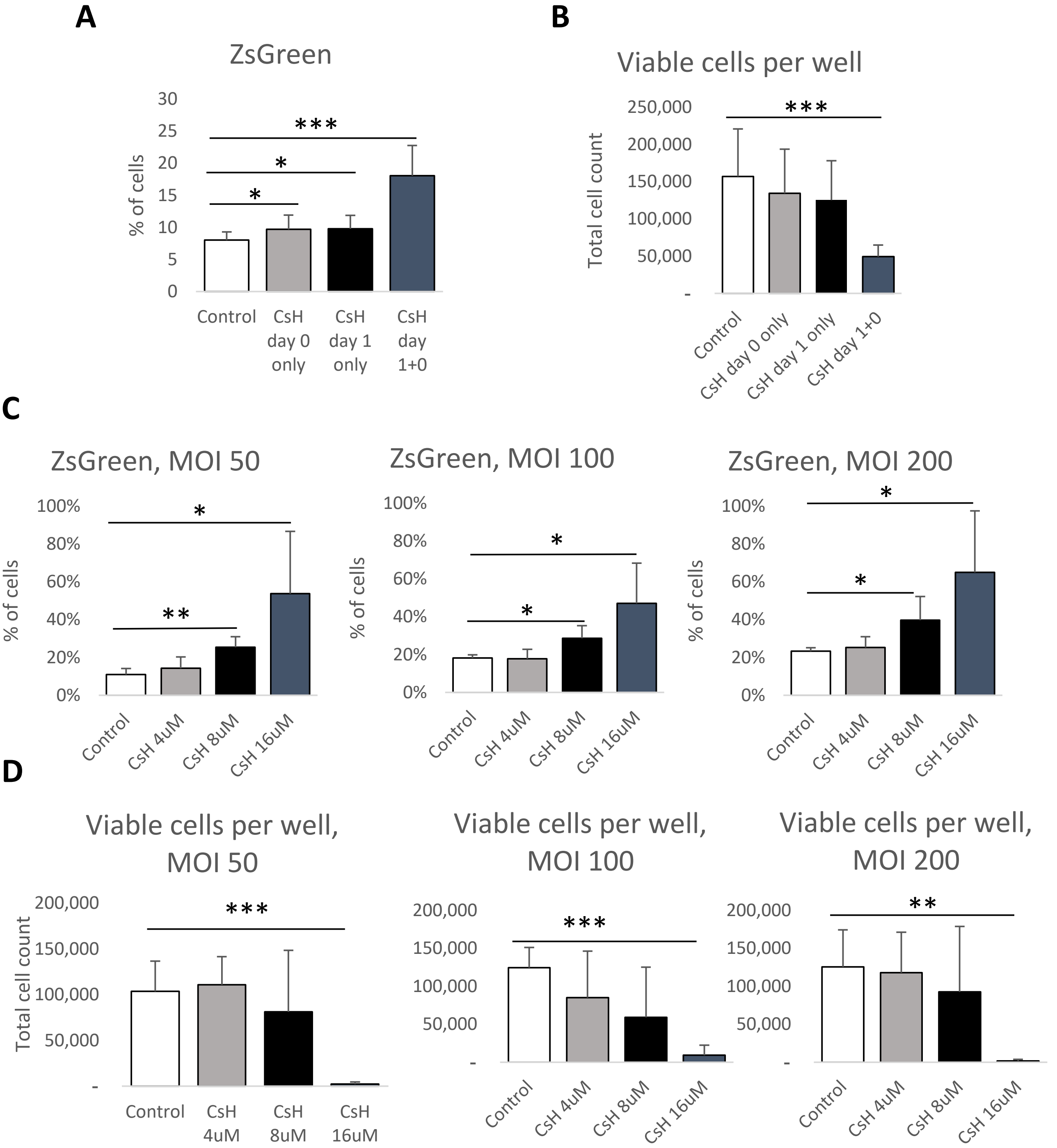

Supplementary Figure S2. Effect of prolonged exposure and various concentrations of CsH on the transduction efficiency and expansion of murine HSPCs. A and **B**. LSK cells were isolated from murine BM, pre-incubated with CsH or DMSO for 24 h (5,000 cells/well), transduced with an LV designed to express ZsGreen fluorescent reporter using spinoculation (MOI 50), incubated with CsH/DMSO for an additional 24 h, and cultured for 6 days. The counts of viable cells and the percentage of cells expressing fluorescent reporter were analyzed using flow cytometry (viable cells were defined as negative for PI stain). Graph (**A**) shows the percentage of cells expressing fluorescent reporter in various experimental groups, while graph (**B**) shows the number of viable cells per well. Data were collected from four independent experiments (n=4). **C** and **D**. LSK cells were sorted from murine BM; 5,000 cells/well were plated and transduced with an LV designed to express ZsGreen fluorescent reporter using spinoculation at MOI 50, 100 or 200, incubated with CsH/DMSO for 24 h, and cultured for 6 days. The counts of viable cells and the percentage of cells expressing fluorescent reporter were determined using flow cytometry (viable cells were defined as negative for PI stain). Part (**C**) shows the percentage of cells expressing fluorescent reporter at various concentrations of CsH for each MOI, while graph (**D**) shows the number of viable cells per well. Data were collected from four independent experiments (n=4). Statistical significance was calculated by an unpaired t-test. *p≤0.05,**p≤0.01, ***p≤0.001.

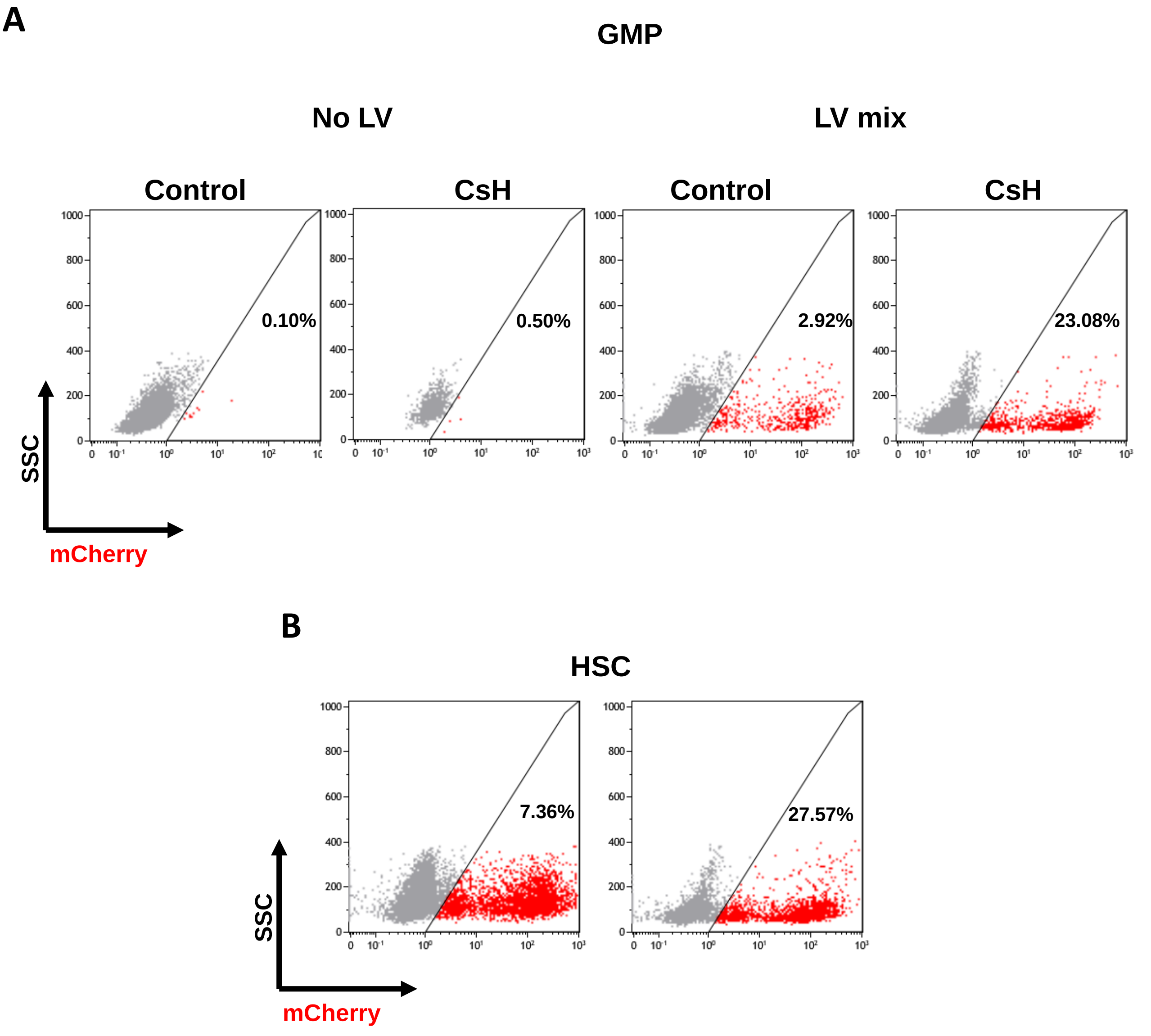

Supplementary Figure S3. CsH increases the efficiency of transduction of GMPs and HSCs in vitro. A. Plots showing the proportion of total mCherry-expressing cells for the data presented in Figure 2. **B.** Plots showing the proportion of total mCherry-expressing cells for the data presented in Figure 3. In this figure, cells were not pre-gated based on the other fluorescent reporters to clearly detect the mCherry signal. The MOI values were 25 for GMPs and 40 for HSCs.
